# Revisiting the Rate-Limiting Step of the ANS–Protein Binding at the Protein Surface and Inside the Hydrophobic Cavity

**DOI:** 10.3390/molecules26020420

**Published:** 2021-01-14

**Authors:** Chikashi Ota, Shun-ichi Tanaka, Kazufumi Takano

**Affiliations:** 1 College of Life Sciences, Ritsumeikan University, Kusatsu, Shiga 525-8577, Japan; 2 Department of Biomolecular Chemistry, Kyoto Prefectural University, Sakyo-ku, Kyoto 606-8522, Japan; stanaka1@kpu.ac.jp

**Keywords:** fluorescence, 8-anilino-1-naphthalenesulfonic acid, protein binding, hydrophobic probe

## Abstract

8-Anilino-1-naphthalenesulfonic acid (ANS) is used as a hydrophobic fluorescence probe due to its high intensity in hydrophobic environments, and also as a microenvironment probe because of its unique ability to exhibit peak shift and intensity change depending on the surrounding solvent environment. The difference in fluorescence can not only be caused by the microenvironment but can also be affected by the binding affinity, which is represented by the binding constant (*K*). However, the overall binding process considering the binding constant is not fully understood, which requires the ANS fluorescence binding mechanism to be examined. In this study, to reveal the rate-limiting step of the ANS–protein binding process, protein concentration-dependent measurements of the ANS fluorescence of lysozyme and bovine serum albumin were performed, and the binding constants were analyzed. The results suggest that the main factor of the binding process is the microenvironment at the binding site, which restricts the attached ANS molecule, rather than the attractive diffusion-limited association. The molecular mechanism of ANS–protein binding will help us to interpret the molecular motions of ANS molecules at the binding site in detail, especially with respect to an equilibrium perspective.

## 1. Introduction

**Figure 1:**
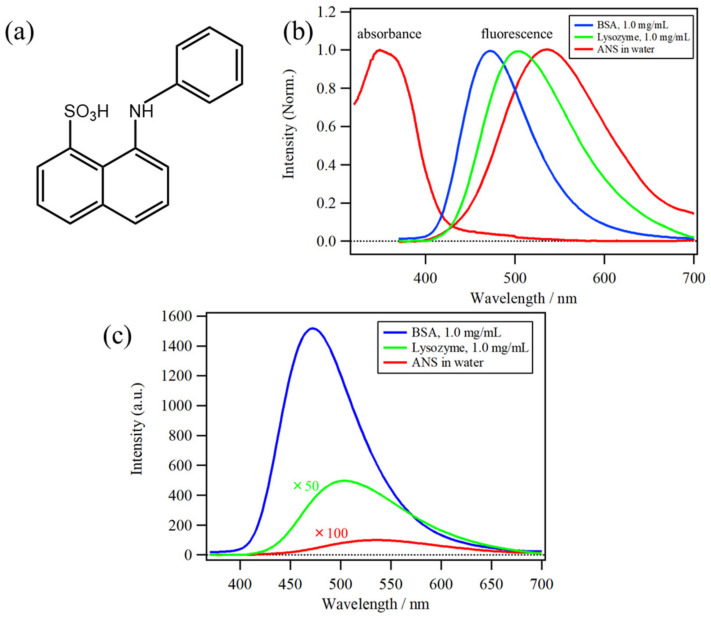
(**a**) Molecular structure of 8-anilino-1-naphthalenesulfonic acid (ANS). (**b**) Normalized absorption and fluorescence spectra of ANS in water, as well as normalized fluorescence spectra solutions of lysozyme (1.0 mg/mL) and bovine serum albumin (BSA) (1.0 mg/mL). (**c**) Comparison of the fluorescence spectrum of BSA (1.0 mg/mL) solution with that of water and lysozyme solution.

**Figure 2:**
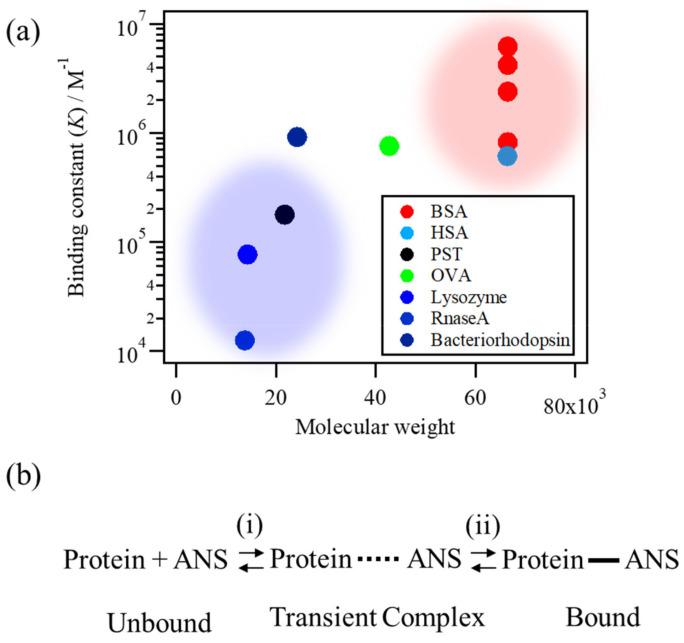
(**a**) The ANS–protein binding constants (*K*) of various proteins [9,10,11,12,13]. (**b**) An assumption about the ANS–protein binding processes.

The chemical compound 8-Anilino-1-naphthalenesulfonic acid (ANS) (Figure 1a) has been used as a hydrophobic fluorescence probe for more than five decades, mainly in biochemical and biological research [1,2,3,4], because of its high fluorescence intensity in hydrophobic environments. ANS has been used to detect protein aggregation and protein denaturation [5,6]. The absorption and fluorescence spectra of ANS in water, as well as the fluorescence in solutions of lysozyme (1.0 mg/mL) and bovine serum albumin (BSA) (1.0 mg/mL), are shown in Figure 1b,c, indicating that both the band position and intensity of ANS fluorescence show significant differences depending on the microenvironments around the binding site of proteins [4,7,8]. Furthermore, a large difference in fluorescence can be caused by the microenvironment and binding affinity (represented by the binding constant, *K*). The binding constants between ANS and proteins from previous studies are plotted in Figure 2a, indicating that the *K* values show a large dispersion by two or three orders of magnitude among proteins [9,10,11,12,13].

A previous thermodynamic study using calorimetric enthalpy measurement [14], however, showed that the binding enthalpies of various proteins, including lysozyme and BSA, do not differ significantly with the main conclusion that ion-pair formation between the negatively charged sulfonate group of ANS and the positively charged amino acid residues of proteins (such as Lys and Arg) is the primary driving force for ANS–protein binding; this has also been elucidated by other protein structural analyses [15,16,17,18]. To clarify the cause of the large dispersion of the binding constants, knowledge of the interaction at the binding site and an understanding of the overall binding process, including the initial process before binding, are essential, requiring a detailed understanding of each component step and its impact.

In the current study, to elucidate the rate-limiting step of the ANS–protein binding process, protein concentration-dependent ANS fluorescence measurements were performed using lysozyme and BSA solutions under various pH conditions. To analyze the binding constant of each condition, we assumed that the overall binding process consists of (i) diffusion-limited association as an initial binding step and (ii) the stabilization process by interaction at the binding site as a latter one through a transient complex state as a representative state appearing in the binding pathways that leads the unbound state to the bound complex (Figure 2b) [19,20]. Based on the aforementioned model, the protein concentration-dependent measurements of ANS fluorescence were analyzed in detail. The effects of the initial and latter binding processes were compared by preparing comparable conditions in the two binding processes. In the initial binding step, the effect of the diffusion-limited association for the overall binding constant was estimated by comparing the ANS–lysozyme binding constants under pH conditions of pH 8.0, 5.5, and 3.0 because the net charge of the lysozyme increases as the pH decreases [21,22], wherein a strong electrostatic attraction between the ANS and lysozyme is expected, which should promote the association. In the latter binding step, the effect of the binding interaction at the binding sites on the protein surface and inside the hydrophobic cavity was estimated by comparing the *K* values of the lysozyme and BSA solutions. Based on the analysis of the experimental results, the free energy surface of the ANS–protein binding was described and the rate-limiting step of the overall process was suggested.

## 2. Results

### 2.1. Molecular Docking Analysis

Before starting the spectroscopic analysis of ANS fluorescence upon binding to proteins, a molecular docking analysis using SwissDock was performed to ascertain the ANS binding site of the proteins [23,24]. Under the protein concentration measurements, in which the amount of protein was excessive compared to that of the ANS molecules, the most preferable binding site had the lowest ΔG.

**Figure 3:**
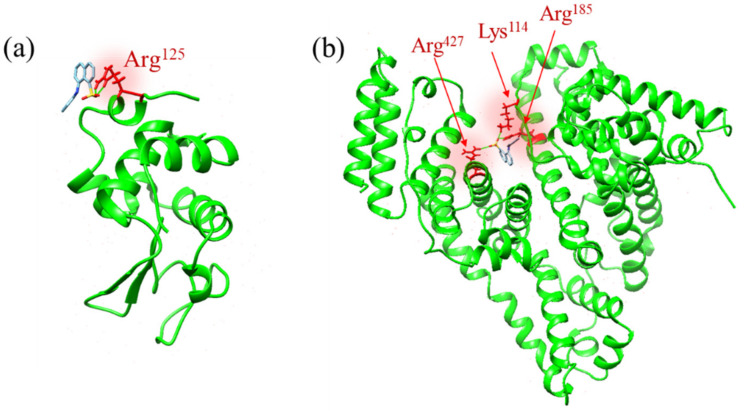
Estimated ANS binding sites of (**a**) lysozyme and (**b**) BSA, which has the lowest ΔG among the various binding sites. (**a**) The binding site of lysozyme is located at Arg^125^ on the protein surface, and (**b**) the binding site of BSA is located inside the hydrophobic pocket with three hydrogen bonds with Lys^114^, Arg^185^, and Arg^427^.

Figure 3a,b show the ANS binding sites of the lysozyme and BSA, which have the lowest ΔG among the various binding sites. The binding site of lysozyme is located at Arg^125^ on the protein surface (Figure 3a), and the binding site of BSA is located inside the hydrophobic pocket with three hydrogen bonds with Lys^114^, Arg^185^, and Arg^427^ (Figure 3b). Previous studies have suggested that ANS molecules can bind to positively charged amino acid residues, such as Lys and Arg, at the protein surface through ion-pair interaction [14,15,16,17,18]. Lysozyme has several Lys and Arg residues on its protein surface [4,25]; thus, Arg^125^ can be the most preferable site for ANS molecules. In contrast, in BSA ANS molecules can be confined inside the hydrophobic pockets of the protein and the principal binding sites are located in the subdomains IIA and IIIA [12,26,27,28]. Here, the estimated binding site in Figure 3b is inside subdomain IIIA; thus, the results acquired by the current simulation agree with the previous results. The estimated ΔG values for the ANS binding site of lysozyme and BSA were −7.1 and −8.6 kcal/mol, respectively, indicating that the ANS binding site of BSA is more stable than that of lysozyme.

### 2.2. Protein Concentration Dependence of ANS Fluorescence

**Figure 4:**
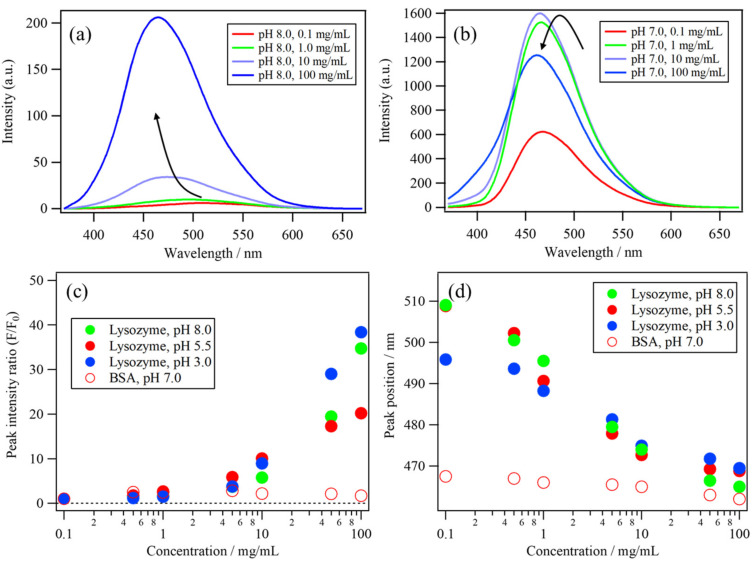
The protein concentration dependences of the ANS fluorescence of (**a**) lysozyme solution (pH 8.0) and (**b**) BSA solution (pH 7.0). The protein concentration dependences of (**c**) the peak intensity ratio (F/F_0_) and (**d**) the peak position of the ANS fluorescence band of lysozyme at pH 8.0, 5.5, and 3.0 and of BSA at pH 7.0.

A protein concentration-dependent measurement of the ANS fluorescence was performed to estimate the binding constant of the ANS–protein binding. To evaluate the binding affinity of the most preferable site, the protein concentration was increased with a lower ANS concentration (30 μM). Figure 4a,b show the lysozyme (pH 8.0) and BSA (pH 7.0) concentration dependences of the ANS fluorescence. The protein concentrations were 0.1, 0.5, 1.0, 5.0, 10, 50, and 100 mg/mL. The ANS fluorescence of the lysozyme solutions gradually increased as the protein concentration increased, indicating the gradual ANS binding to the lysozyme molecule (Figure 4a). The ANS fluorescence of the BSA solutions immediately increased after 0.1 mg/mL, implying a high affinity between ANS and BSA (Figure 4b). On the other hand, the fluorescence decreased after 1.0 mg/mL because of the decrease in the throughput of the excitation light at 350 nm, which could be caused by increased absorbance in the near-UV region of BSA [4].

Figure 4c shows the protein concentration dependences of the peak intensity ratio (F/F_0_) and Figure 4d shows the peak position of the ANS fluorescence band of lysozyme solutions at pH 8.0, 5.5, and 3.0, as well as BSA at pH 7.0. Both the peak intensity ratio and its position in the lysozyme solutions gradually changed as the concentration increased. In contrast, those of BSA did not show a significant change because the ANS fluorescence intensity and the position of the BSA solution immediately changed at lower concentrations. To understand these changes in the ANS fluorescence of the lysozyme and BSA solutions in detail, principal component analysis (PCA) and multivariate curve resolution-alternating least squares (MCR–ALS) were applied to these datasets, as described in the subsequent section [29,30,31,32].

### 2.3. MCR–ALS Analysis of the Protein Concentration Dependence of ANS Fluorescence Spectra

The number of components in the datasets of the lysozyme concentration dependence of ANS fluorescence at pH 8.0, 5.5, and 3.0 was analyzed by an eigenvalue plot using PCA, which then indicated that the datasets mainly had two components in these concentration ranges. Thus, based on the two-component approximation, MCR–ALS was applied to resolve the spectral datasets.

**Figure 5:**
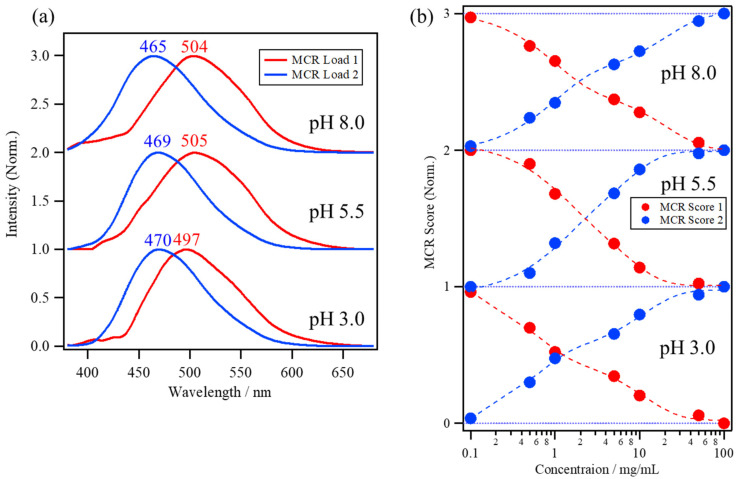
(**a**) The multivariate curve resolution (MCR) loadings and (**b**) scores of the ANS fluorescence spectra of lysozyme solutions at pH 8.0, 5.5, and 3.0.

Figure 5a shows the MCR loadings and Figure 5b shows the scores of the ANS fluorescence spectra of the lysozyme solutions at pH 8.0, 5.5, and 3.0. The first MCR loading (red line) has a red-shifted peak maximum at approximately 500 nm compared to the second MCR loading (blue line) with a peak maximum at approximately 470 nm. The first MCR loading can be assigned to free ANS molecules that are diffusing in water or that are a part of the ANS molecules binding to lysozyme because ANS molecules in hydrophilic environments have a red-shifted weak fluorescence from the charge-transfer (CT) electronic state, which transitions from the non-planar (NP) excited state with the rotation of the phenylamino group [2,4,18,33]. The second MCR loading, with a blue-shifted peak at approximately 470 nm, can be assigned to the ANS molecules binding to the protein surface, because both the restriction of water reorientation for ANS molecules in the protein hydration layer [4,34,35] and the steric restriction of the ANS conformation at the binding site [12,36] can prevent transition from the NP state to the CT state, resulting in a blue-shift in the ANS fluorescence. The scores belonging to MCR Score 1 of the lysozyme solutions at pH 8.0, 5.5, and 3.0 (red circle) in Figure 5b show a decrease with an increase in the protein concentration, while those of MCR Score 2 (blue circle) show an increase with an increase in concentration.

**Figure 6:**
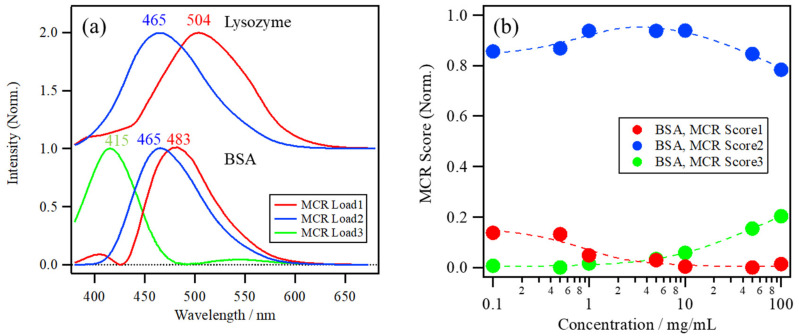
(**a**) The MCR loadings and (**b**) scores of the ANS fluorescence spectra of the BSA solution at pH 7.0, with those of the lysozyme solution at pH 8.0 as a reference.

The number of components in the datasets of the BSA concentration dependence of ANS fluorescence at pH 7.0 was analyzed by the eigenvalue plot using PCA, which then indicated that the datasets had mainly three components in this concentration range. Thus, based on the three-component approximation, MCR–ALS was applied to resolve the spectral dataset. Figure 6a shows the MCR loadings and Figure 6b shows the scores of the ANS fluorescence spectra of the BSA solution at pH 7.0 in the lower portion, with those of the lysozyme solution at pH 8.0 as a reference in the upper portion. The first, second, and third MCR loadings have peak maxima at around 483, 465, and 415 nm, respectively. The first MCR loading, with a peak at 483 nm, is blue-shifted compared to that in the lysozyme solution at 504 nm, implying that a large number of ANS molecules were already bound to BSA in the lower concentration range because of its high affinity with ANS molecules. The second loading is much more blue-shifted compared to the first, and is located at the same peak position as the lysozyme, which can be assigned to the ANS molecules binding to the hydrophobic pockets of BSA. The third loading, with a peak at 415 nm, can be assigned to the intrinsic protein fluorescence at the oligomeric interface of BSA oligomers because of the delocalization of the electrons of protein backbones at the interface by the weak hydrogen bond and/or π–π stacking interactions [4,31,37,38].

MCR Score 1 of the BSA solution in Figure 6b was only a small part of the overall score and shows a gradual decrease with an increase in the protein concentration, while MCR Score 2 is dominant throughout the entire concentration range, especially at >1.0 mg/mL, indicating the high binding affinity of ANS to BSA. On the other hand, MCR Score 3 starts to increase in the higher concentration range after 10 mg/mL, caused by the intrinsic fluorescence induced by BSA oligomerization. MCR Score 3 is not directly related to the ANS binding. Therefore, only MCR Scores 1 and 2 have been focused on for the analysis of the ANS–protein binding constant.

### 2.4. Estimation of the ANS–Protein Binding Constant

To compare the binding affinity of ANS to proteins of each protein solution, the binding constant was estimated based on the following assumption of the equilibrium relation [39,40]:

(1) $$K=\frac{C_{R}\left(MCR2\right)}{C_{R}\left(MCR1\right)\left[Protein\right]},$$

where *C_R_(MCR1)* and *C_R_(MCR2)* are the normalized MCR scores 1 and 2 and *[Protein]* is the protein concentration of the lysozyme and BSA solutions. Based on the assumption shown above, the binding constant can be estimated using Equation (2) [39,40]:

(2) $$C_{R}\left(MCR2\right)=\frac{K\left[Protein\right]}{1+K\left[Protein\right]}.$$

It should be noted that the protein concentration is excessive compared to the ANS concentration (30 μM) in the protein concentration-dependent measurements; thus, the estimated binding constant can be assigned to the most preferable binding site of the overall protein surface.

**Figure 7:**
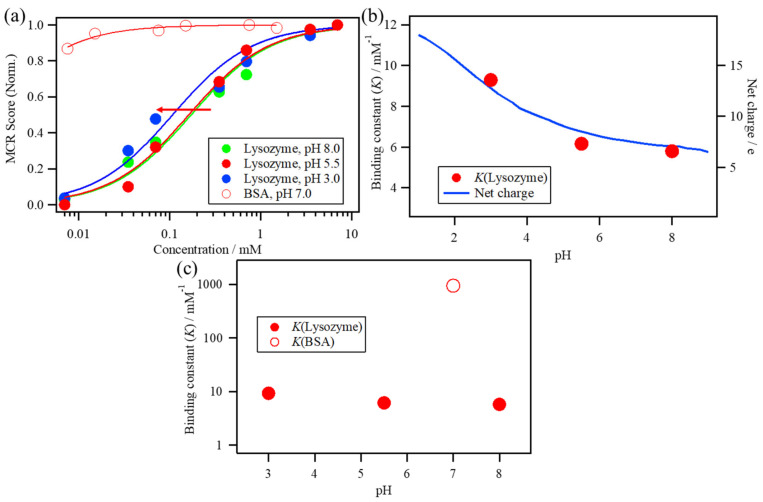
(**a**) The increase in MCR Score 2 of the lysozyme solutions at pH 8.0, 5.5, and 3.0 and the BSA solution plotted against the protein concentration. (**b**) The estimated *K* value of the lysozyme solutions at pH 8.0, 5.5, and 3.0 with the calculated net charge of lysozyme as a function of pH. (**c**) The estimated *K* value of the ANS–BSA binding with one of the lysozyme solutions.

The fitting results and the estimated *K* values of these conditions are shown in Figure 7. The increase in the MCR Score 2 of the lysozyme solutions shifts to a lower concentration as the pH of the solutions decreases from 8.0 to 3.0 (Figure 7a). The estimated *K* values of the lysozyme solutions at pH 8.0, 5.5, and 3.0 correspond to 5.8, 6.2, and 9.3 mM^−1^, respectively, indicating that the estimated *K* values of the lysozyme solutions at pH 8.0, 5.5, and 3.0 slightly increase as the pH decreases and that the *K* value at pH 3.0 is approximately 1.6 times larger than that at pH 8.0 (Figure 7b). The net charge of the lysozyme was estimated as a function of pH from the number of individual acidic and basic amino acid residues and their corresponding acidity constants using the web server H++ at http://biophysics.cs.vt.edu/index.php [21,22,41]. The net charge of the lysozyme increases as pH decreases because of the protonation of amino acid residues. It should be noted that the immediate increase in the net charge from pH 5.5 to pH 3.0 is related to the increase in the *K* value of the lysozyme solutions, suggesting that the increase in the net charge of the lysozyme can promote ANS binding to the lysozyme.

The increase in MCR Score 2 of BSA has a high score value even at lower concentrations because of its high affinity (Figure 7a). The estimated *K* value of BSA corresponds to 940 mM^−1^, which is higher than that of lysozyme by two orders of magnitude and is 160 times larger than that of lysozyme at pH 8.0, as shown in Figure 7c.

## 3. Discussion

### 3.1. Effect of the Electrostatic Association on the ANS–Protein Binding Pathway

To estimate the effect of the diffusion-limited association as well as the binding interaction at the binding site, the protein concentration dependence of ANS was measured using lysozyme and BSA solutions as a case study. Lysozyme concentration-dependent measurements of ANS fluorescence at various pH values were performed to estimate the binding constant, indicating that the *K* value of lysozyme increased by 1.6 times from pH 8.0 to pH 3.0. The pH-dependent increase in the *K* value can be caused by the electrostatically assisted association between the negatively charged sulfonate group of ANS and the increasing positively charged lysozyme with the decrease in pH of the solutions.

Previous studies have shown an electrostatic association by accelerated diffusion in the binding interfaces of protein complexes [19,42,43]. Schreiber and Fersht showed a rapid, electrostatically assisted association between barnase and its intracellular inhibitor barstar using the electrostatic screening effect of NaCl [42]. The binding site of barnase is positively charged, whereas that of barstar has complementary negative charges, causing electrostatically assisted association at the oppositely charged interface. They showed a linear correlation between the association rate constant (k_1_) and the electrostatic contribution to the mean rational activity coefficient of NaCl ($f_{\pm }^{*}$), which was calculated using the Debye–Hückel equation. This suggests that the rate-limiting step for an association is dictated mainly by the electrostatic potential between the two proteins, because log $f_{\pm }^{*}$ is directly related to the electrostatic potential and log k_1_ is directly related to the activation energy. The association rate constant was changed from 5 × 10^9^ to 10^5^, a change of order corresponding to NaCl concentrations ranging from 5 mM to 2000 mM.

The association rate constant between barnase and barstar with NaCl concentrations from 2000 mM to 5 mM increased by four orders of magnitude, while the binding constant between ANS and lysozyme increased by 1.6 times from pH 8.0 to pH 3.0, suggesting that a rapid electrostatically assisted association cannot be achieved in ANS–lysozyme binding because the fraction of the reaction area (binding interface) was only a small portion of the overall protein and is much smaller compared to that associated with the case of the association between barnase and barstar. Therefore, in ANS–lysozyme binding, the acceleration effect due to the electrostatic association can only contribute a small part to the diffusion-limited association.

### 3.2. Effects of the Binding Interaction on the ANS–Protein Binding Pathway

Comparison of the estimated *K* value for the ANS–BSA binding with that of lysozyme (Figure 7c) showed that the estimated *K* value of BSA was much higher than that of lysozyme by two orders of magnitude. One of the critical factors behind the high affinity of BSA could be expected by the binding enthalpy at the binding site because the binding enthalpy primarily reflects the strength of the interaction between ANS and the bound protein. Matulis and Lovrien estimated the enthalpies of binding ANS to four proteins (BSA, lysozyme, papain, and protease omega). They showed that the ANS binding enthalpies of one site in proteins have −4 to −5 kcal/mol (−17 to −21 kJ/mol), whether the binding site is on the protein surface or inside the hydrophobic pocket, suggesting that the primary factor in the binding of ANS to proteins is electrostatic (Coulombic) interaction through ion-pair formation at the binding site [14]. In other words, the binding enthalpies hardly depend on the hydrophobic or hydrophilic environment surrounding the binding site. Therefore, the difference in *K* value between BSA and lysozyme cannot be explained from the perspective of binding enthalpy at the binding site.

Previous studies of the dissociation constant of ANS–protein binding using the ANS concentration dependence measurement show that the dissociation constant of BSA has a smaller value of a few micromolars, such as 1.3 [44] or 5.0 μM [26], compared to those of external sites, such as MurA (40.8 μM) [16], Poly-Arg (2.9 mM), and Poly-Lys (2.6 mM) [18], suggesting that the low dissociation constant can be caused by the hydrophobic pocket of BSA. This supports the results of the present study, showing that the binding constant, the *K* value of ANS–BSA binding, was much higher than that of ANS–lysozyme binding, in which the binding site is at the external site of proteins. Therefore, it can be suggested that the binding of ANS inside the hydrophobic pocket can restrict the dissociation of ANS from the binding site because of its confined environment, thereby increasing the *K* value.

### 3.3. Rate-Limiting Step of the Overall ANS–Protein Binding Pathway

**Figure 8:**
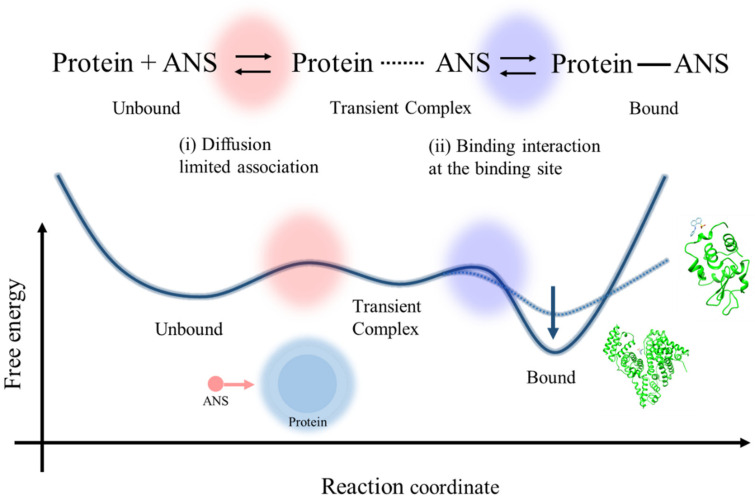
The proposed free energy surface of the ANS–protein binding with the kinetic scheme of the association between ANS and protein molecules via the formation of the transient complex.

Based on the protein concentration dependence on ANS fluorescence using lysozyme and BSA solutions, the proposed free energy surface of the ANS–protein binding with the kinetic scheme of the association between ANS and protein molecules via the formation of the transient complex is shown in Figure 8. The ANS–protein binding can be classified as the interaction between a macromolecule and a small binding molecule; thus, the fraction corresponding to the active site is only a small part of the overall protein. Therefore, the diffusion-limited association process between the unbound state and transient complex state (red-colored part) has a small contribution to the overall binding process, as is experimentally shown in the small effect of the electrostatic acceleration by the increased net charge of the protein in the effect of pH on the ANS–lysozyme binding constant.

On the other hand, the latter process, after the initial binding (blue-colored part), can be rather crucial because the microenvironment around the binding site has a significant effect on the overall binding constant by stabilizing the binding state and preventing the dissociation process, as is evident experimentally in the comparison of the *K* values of ANS–BSA binding in the case of lysozyme. The confined environment in the hydrophobic pockets of BSA can decrease the free energy of the bound state compared to the case of lysozyme, as shown in the molecular docking simulation (Figure 3). It can increase the potential barrier from the bound state to the transient complex state, as indicated by the small value of the dissociation constant of BSA in previous studies [26,44].

Therefore, it can be suggested that the binding interaction at the binding site is the main factor affecting the binding constant of the overall ANS–protein binding process. The latter process from the transient complex state to the bound state is the main limiting step.

## 4. Experimental Section

### 4.1. Fluorescence Measurement

A protein concentration-dependent measurement of ANS fluorescence was performed to estimate the binding constant of ANS–protein binding. To evaluate the binding affinity of the most preferable site, the protein concentration was increased with a lower ANS concentration (30 μM). The protein concentrations of the lysozyme and BSA solutions were prepared at 0.1, 0.5, 1.0, 5.0, 10, 50, and 100 mg/mL. To investigate the effect of the diffusion-limited association on the overall binding constant, lysozyme solutions were prepared at pH 8.0, 5.5, and 3.0; citrate buffer solution (20 mM) was used to establish pH 5.5 and 3.0, and Tris-HCl buffer solution (20 mM) was used to establish pH 8.0. To compare the binding constants between lysozyme and BSA, the BSA solution was prepared at pH 7.0 with 20 mM of citrate buffer solution to evaluate the effect of the interaction at the binding site. 

The experimental setup for the measurement of fluorescence spectra has been previously described [4,7]. ANS fluorescence spectra were measured in a quartz cuvette using an F-2500 system (Hitachi, Ltd., Tokyo, Japan) with a right-angle geometry. The excitation wavelength was set to 350 nm and the spectral range was from 300 to 800 nm in 0.5 nm increments.

### 4.2. Molecular Docking Analysis

To estimate the ANS binding sites of lysozyme and BSA, a molecular docking analysis was carried out, using SwissDock at http://www.swissdock.ch, which is a protein-small molecule docking web server [23,24]. SwissDock achieves a high success rate of the biding site prediction compared to other well-known protein docking tools [45]. SwissDock estimates the preferable binding sites of proteins based on the CHARMM22 force field calculating the van der Waals and electrostatic interaction energy between the ligand and the target protein. In addition, the solvent effect is taken into account using the FACTS (Fast Analytical Continuum Treatment of Solvation) implicit solvation model, which implicitly considers the solvent effect under the assumption of the continuum electrostatics models.

## 5. Conclusions

To clarify the rate-limiting step of the ANS–protein binding process, protein concentration-dependent measurements of ANS fluorescence were performed using lysozyme and BSA solutions. The diffusion-limited association process in the initial binding, which can be accelerated by the electrostatic association in the lower pH condition, has a small effect on the overall binding constant because the fraction corresponding to the binding site is only a small part of the overall protein. In contrast, after the initial binding the latter process has a larger effect on the binding constant, especially in the case of the binding site being in hydrophobic pockets, which prevents the dissociation of ANS from the site due to the confined environment. The results of this study suggest that the main factor affecting the ANS–protein binding process is the microenvironment at the binding site, via the restriction of the attached ANS molecule rather than the attractive diffusion-limited association between ANS and the protein. The molecular mechanism of ANS–protein binding will help us to interpret the molecular motions of ANS molecules at the binding site in detail, especially from the perspective of equilibrium.
